# Neuronal CCL2 is upregulated during hepatic encephalopathy and contributes to microglia activation and neurological decline

**DOI:** 10.1186/1742-2094-11-121

**Published:** 2014-07-10

**Authors:** Matthew McMillin, Gabriel Frampton, Michelle Thompson, Cheryl Galindo, Holly Standeford, Eric Whittington, Gianfranco Alpini, Sharon DeMorrow

**Affiliations:** 1 Department of Internal Medicine, Texas A&M Health Science Center, College of Medicine, 1901 South 1st Street, Building 205, Temple, Texas, USA; 2 Digestive Disease Research Center, Scott & White Hospital, 1901 South 1st Street, Building 205, Temple, Texas, USA; 3 Central Texas Veterans Healthcare System, 1901 South 1st Street, Building 205, Temple, Texas, USA

**Keywords:** Acute liver failure, Azoxymethane, CCR2, CCR4, Neuroinflammation

## Abstract

**Background:**

Acute liver failure leads to systemic complications with one of the most dangerous being a decline in neurological function, termed hepatic encephalopathy. Neurological dysfunction is exacerbated by an increase of toxic metabolites in the brain that lead to neuroinflammation. Following various liver diseases, hepatic and circulating chemokines, such as chemokine ligand 2 (CCL2), are elevated, though their effects on the brain following acute liver injury and subsequent hepatic encephalopathy are unknown. CCL2 is known to activate microglia in other neuropathies, leading to a proinflammatory response. However, the effects of CCL2 on microglia activation and the pathogenesis of hepatic encephalopathy following acute liver injury remain to be determined.

**Methods:**

Hepatic encephalopathy was induced in mice via injection of azoxymethane (AOM) in the presence or absence of INCB 3284 dimesylate (INCB), a chemokine receptor 2 inhibitor, or C 021 dihydrochloride (C021), a chemokine receptor 4 inhibitor. Mice were monitored for neurological decline and time to coma (loss of all reflexes) was recorded. Tissue was collected at coma and used for real-time PCR, immunoblots, ELISA, or immunostaining analyses to assess the activation of microglia and consequences on pro-inflammatory cytokine expression.

**Results:**

Following AOM administration, microglia activation was significantly increased in AOM-treated mice compared to controls. Concentrations of CCL2 in the liver, serum, and cortex were significantly elevated in AOM-treated mice compared to controls. Systemic administration of INCB or C021 reduced liver damage as assessed by serum liver enzyme biochemistry. Administration of INCB or C021 significantly improved the neurological outcomes of AOM-treated mice, reduced microglia activation, reduced phosphorylation of ERK1/2, and alleviated AOM-induced cytokine upregulation.

**Conclusions:**

These findings suggest that CCL2 is elevated systemically following acute liver injury and that CCL2 is involved in both the microglia activation and neurological decline associated with hepatic encephalopathy. Methods used to modulate CCL2 levels and/or reduce CCR2/CCR4 activity may be potential therapeutic targets for the management of hepatic encephalopathy due to acute liver injury.

## Introduction

Hepatic encephalopathy is a serious neuropsychiatric complication of both acute liver failure and chronic liver diseases with the potential to affect health-related quality of life, clinical management strategies, priority for liver transplantation, and patient survival [1]. The etiology and progression of hepatic encephalopathy due to acute liver failure and chronic liver diseases differs significantly. For example, hepatic encephalopathy resulting from acute liver failure causes altered mental states and cognitive disruptions that can progress to coma in hours or days [2]. Conversely, during cirrhosis, hepatic encephalopathy develops slowly, with many patients having altered sleep patterns and cognitive issues which can progress to more severe symptomology if no therapeutic intervention is given [3]. Associated with these differences in neurological presentation, the disease processes that generate hepatic encephalopathy due to acute and chronic liver failure differ significantly. For example, the presence of cerebral edema with elevated intracranial pressure is only observed in patients with acute liver failure [4]. However, the one pathological process common to hepatic encephalopathy that is independent of the type or longevity of liver insult is the activation of neuroinflammatory cascades.

A substantial body of evidence exists suggesting a role for proinflammatory mechanisms in the progression of hepatic encephalopathy. In patients and in animal models of hepatic encephalopathy, systemic inflammation causes worsening of the encephalopathy, and proinflammatory signals may act synergistically with ammonia toxicity to bring about the neurological complications of acute and chronic liver failure [1]. Indeed, tumor necrosis factor-α, interleukin (IL)-1β, and IL-6 levels were increased in patients with acute liver failure [5] as well as in rodent models of acute liver failure [6], and are thought to contribute to cerebral edema and intracranial pressure. In addition, the number of activated microglia is increased in rodent models of hepatic encephalopathy and the use of anti-inflammatory agents, such as minocycline, inhibit the microgliosis and delay the onset of neurological symptoms [7].

In addition to the proinflammatory cytokines, neuroinflammation can be regulated by chemokines or chemotactic cytokines. They are involved in cell-cell communication, effecting a directional migration and activating various cell types of the immune system. In the brain, the chemokine ligand 2 (CCL2 or monocyte chemotactic protein-1) and its receptors chemokine receptor 2 (CCR2) and chemokine receptor 4 (CCR4) have been implicated in a number of neuropathologies ranging from traumatic brain injury to autoimmune diseases [8]. CCL2 can be produced by a number of cell types in the brain, including neurons [8,9]. The consequences of CCL2 expression are varied and context-dependent. For example, CCL2 has been shown to activate microglia as well as increase the infiltration of circulating macrophages [8]. Furthermore, while CCL2 expression is upregulated in a number of neuropathies, the consequences are detrimental in some disease states and protective in others [8].

At this time, no data exist concerning the role of chemokines in the progression of hepatic encephalopathy due to acute liver failure. Therefore, the aims of this study were to assess the expression of CCL2 and its receptors in a rodent model of hepatic encephalopathy due to acute liver failure and to determine how CCL2 contributes to the neurological decline observed in this disorder. This may help identify one of the signals involved in initiating the proinflammatory response that occurs during hepatic encephalopathy.

## Methods

### Materials

Antibodies against IBA1 were purchased from Wako Chemicals USA (Richmond, VA, USA). Antibodies against CCL2, CCR2, and CCR4 were purchased from Genetex (Irvine, CA, USA). NeuN antibodies were ordered from Millipore (Billerica, MA, USA). All real-time PCR (RT-PCR) primers were purchased from SABiosciences (Frederick, MD, USA). INCB 3284 dimesylate (INCB) and C021 dihydrochloride (C021) were purchased from Tocris Bioscience (Minneapolis, MN, USA). All other chemicals were purchased from Sigma-Aldrich (St. Louis, MO, USA) unless otherwise noted, and were of the highest grade available.

### Mouse model of hepatic encephalopathy

All *in vivo* experiments were performed in male C57Bl/6 mice (20 to 25 g; Charles River Laboratories, Wilmington, MA, USA). Mice were given free access to water and rodent chow and were housed in constant temperature, humidity, and 12 h light-dark cycling. Acute liver failure was induced via a single intraperitoneal (ip) injection of 100 mg/kg of azoxymethane (AOM). In parallel, systemic inhibition of CCR2 and CCR4 activity was accomplished via pretreatment with INCB (1 mg/kg/day ip) or C021 (1 mg/kg/day ip) for 3 days prior to injection of AOM. Following injection, mice were placed on heating pads adjusted to 37°C and monitored frequently for signs of neurological decline. To reduce the impacts of hypoglycemia and dehydration, cage floors were supplied with hydrogel and rodent chow and after 12 h, and every subsequent 4 h, mice were injected subcutaneously with 5% dextrose in 250 μL of saline. If mice underwent a 20% or greater weight loss they were removed from the study. All animal experiments performed were approved by and complied with the Baylor Scott & White IACUC regulations on animal experiments (protocol #2011-052-R).

At 8 h following injection (and every 2 h after), body temperature, weight, and neurological assessments were measured. Neurological functioning was assessed by measuring the pinna reflex, corneal reflex, tail flexion reflex, escape response reflex, righting reflex, and ataxia, which were assessed and scored on a scale of 0 (no reflex) to 2 (intact reflex). The neurological score at each time point was defined as the summation of these reflex scores. In addition, time to coma (defined as a loss of all reflexes) was recorded. Tissue was flash frozen and collected at coma (loss of corneal and righting reflexes) for further analysis. Mice used for histochemical studies were transcardially perfused with PBS followed by 4% paraformaldehyde. Whole brains were removed and placed into paraformaldehyde for 24 h, after which they were moved to a 30% sucrose solution for cryoprotection. Brains were frozen and sectioned using a cryostat for immunofluorescence imaging.

### Liver histology and biochemistry

Paraffin-embedded livers were sectioned into 3 μm sections and mounted onto positively charged slides (VWR, Radnor, PA, USA). Slides were deparaffinized and stained with Hematoxylin QS (Vector Laboratories, Burlingame, CA, USA) for 1 min followed by staining for 1 min with eosin Y (Amresco, Solon, OH, USA) and rinsed in 95% ethanol. The slides were then dipped into 100% ethanol and subsequently through two xylene washes. Coverslips were mounted onto the slides using Vectamount mounting media (Vector Laboratories). The slides were viewed and imaged using an Olympus BX40 microscope with an Olympus DP25 imaging system (Olympus, Center Valley, PA, USA).

Serum alanine aminotransferase (ALT) and bilirubin were assessed using commercially available kits. ALT measurement was performed using a fluorometric activity assay (Sigma-Aldrich). Total bilirubin was assayed using a total bilirubin ELISA (CusaBio, Wuha, China). All assays and subsequent analyses were performed according to manufacturers’ instructions.

### Real-time PCR

RNA was extracted from flash frozen tissue and RT-PCR was performed as previously described [10], using commercially available primers designed against mouse CCL2, IL-1β, IL-6, and glyceraldehyde 3-phosphate dehydrogenase. A ΔΔCT analysis was performed using vehicle-treated tissue or untreated primary neurons as controls for subsequent experiments [11,12]. Data for all experiments are expressed as mean relative mRNA levels ± SEM. The sample size for each experiment in each case is indicated in the figure legends.

### Immunoblotting

Immunoblots were performed as previously described [13] with minor modifications. For western blots, 10% sodium dodecyl sulfate-polyacrylamide gel electrophoresis gels were loaded with 10 to 20 μg of protein diluted in Laemmli buffer. Specific primary antibodies against CCL2, CCR2, CCR4, phosphorylated extracellular signal-regulated kinase 1/2 (pERK1/2), total extracellular signal-regulated kinase 1/2 (tERK1/2), and β-actin were used along with appropriate fluorescent secondary antibodies (LI-COR, Lincoln, NE, USA). All imaging was performed on an Odyssey 9120 Infrared Imaging System (LI-COR). Data are expressed as fold change in fluorescent band intensity of target antibody divided by β-actin or tERK1/2, which were used as loading controls. The values of vehicle or control groups were used as a baseline and set to a relative protein expression value of 1. All treatment groups were represented as changes of fluorescent band intensity of target antibody to β-actin or tERK1/2 relative to vehicle or control groups. All band intensity quantifications were performed using ImageJ software (National Institutes of Health, Bethesda, MD, USA). Data for all experiments were expressed as mean relative protein ± SEM (n = 3).

### Immunofluorescence

Free-floating immunostaining was performed on brain sections using anti-IBA1 immunoreactivity to detect the morphology and relative staining of microglia. In addition, CCL2 and NeuN immunostaining were performed. Immunoreactivity was visualized using fluorescent secondary antibodies labeled with Dylight 488 or Cy3 and counterstained with ProLong^©^ Gold Antifade Reagent containing 4’,6-diamidino-2-phenylindole (DAPI). Slides were viewed and imaged using a Leica TCS SP5-X inverted confocal microscope (Leica Microsystems, Buffalo Grove, IL, USA). Quantification of photomicrographs was performed by converting images to grayscale, inverting their color and quantifying field staining intensity with ImageJ software.

### Statistical analysis

All statistical analyses were performed using Graphpad Prism software (Graphpad Software, La Jolla, CA, USA). Results were expressed as mean ± SEM. For data that passed normality tests, significance was established using the Student’s *t*-test when differences between two groups were analyzed, and analysis of variance when differences between three or more groups were compared followed by the appropriate post hoc test. If tests for normality failed, two groups were compared with a Mann-Whitney U test or a Kruskal-Wallis ranked analysis when more than two groups were analyzed. Differences were considered significant when the *P* value was less than 0.05.

## Results

### Microglia activation occurs during AOM-induced hepatic encephalopathy

**Figure 1:**
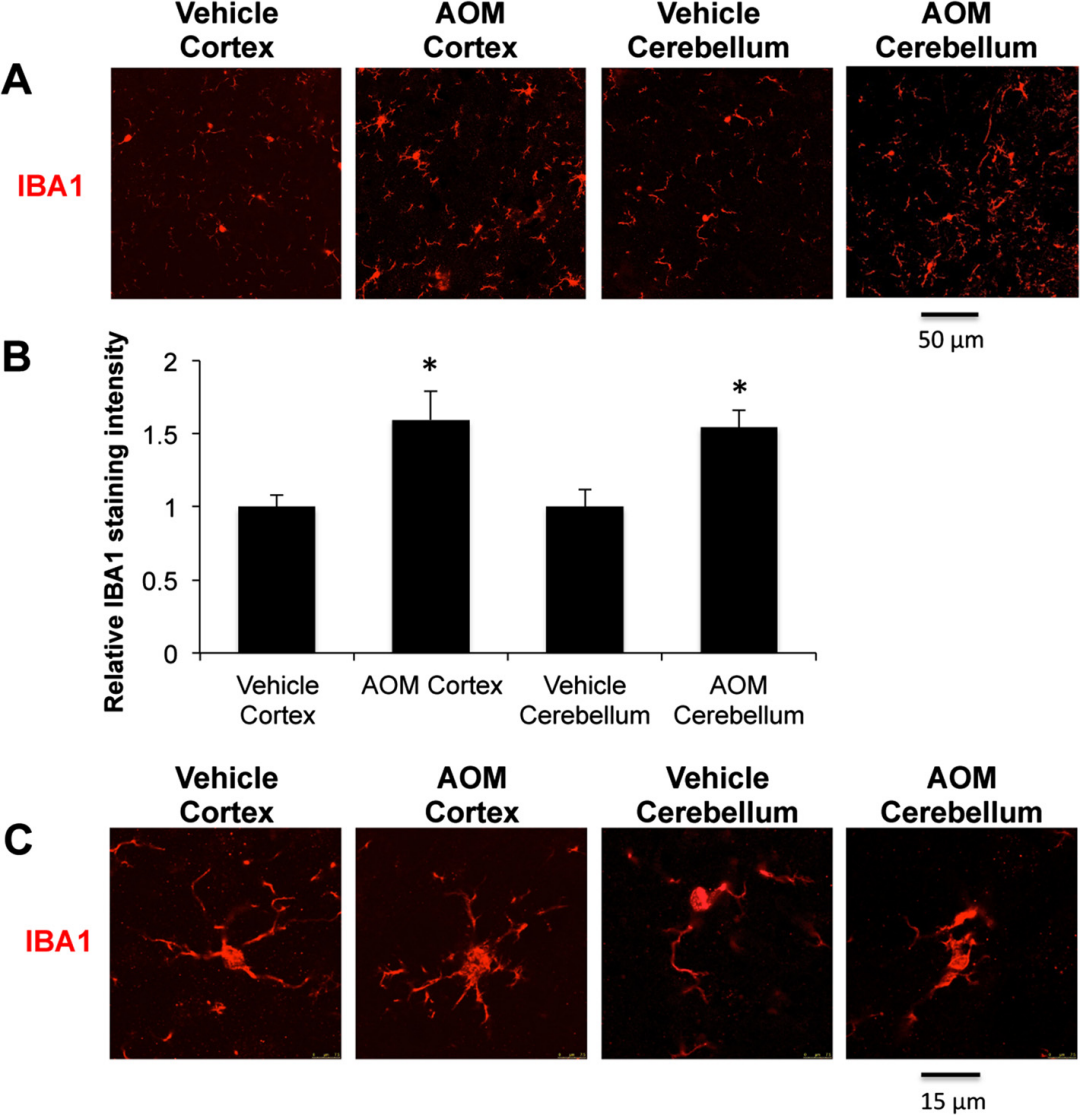
**Microglia activation occurs globally in the brain during hepatic encephalopathy. (A)** Field IBA1 field fluorescence (red) in the cortex and cerebellum from vehicle and AOM-treated mice. **(B)** Quantification of IBA1 field fluorescence in the cortex and cerebellum from vehicle- and AOM-treated mice. **(C)** IBA1 immunofluorescence (red) in individual cells in vehicle cortex, AOM cortex, vehicle cerebellum, and AOM cerebellum. For immunofluorescence quantification analyses * *P* <0.05 compared to vehicle-treated mice, n = 3.

Liver damage leading to the development of hepatic encephalopathy has been previously demonstrated to be dependent upon increased microglia activation and subsequent neuroinflammation [7,14]. Therefore, microglia proliferation and activation were assessed in the brains of AOM-treated mice. Free-floating immunofluorescence was performed using the microglia marker IBA1. AOM-treated mice had a significant increase in IBA1 fluorescence intensity in both the cortex and cerebellum compared to vehicle-treated mice, indicative of microgliosis, which in this study is defined as microglia aggregation in response to injury or stress and is indicative of a neuroinflammatory state (Figure 1A,B). In order to see if individual microglia have a shift in their morphology, IBA1 fluorescence was assessed in the cortex and cerebellum, identifying that microglia have a more amoeboid appearance with retracted processes in AOM-treated mice compared to vehicle-treated mice (Figure 1C).

### CCL2 is elevated following AOM-induced liver damage

**Figure 2:**
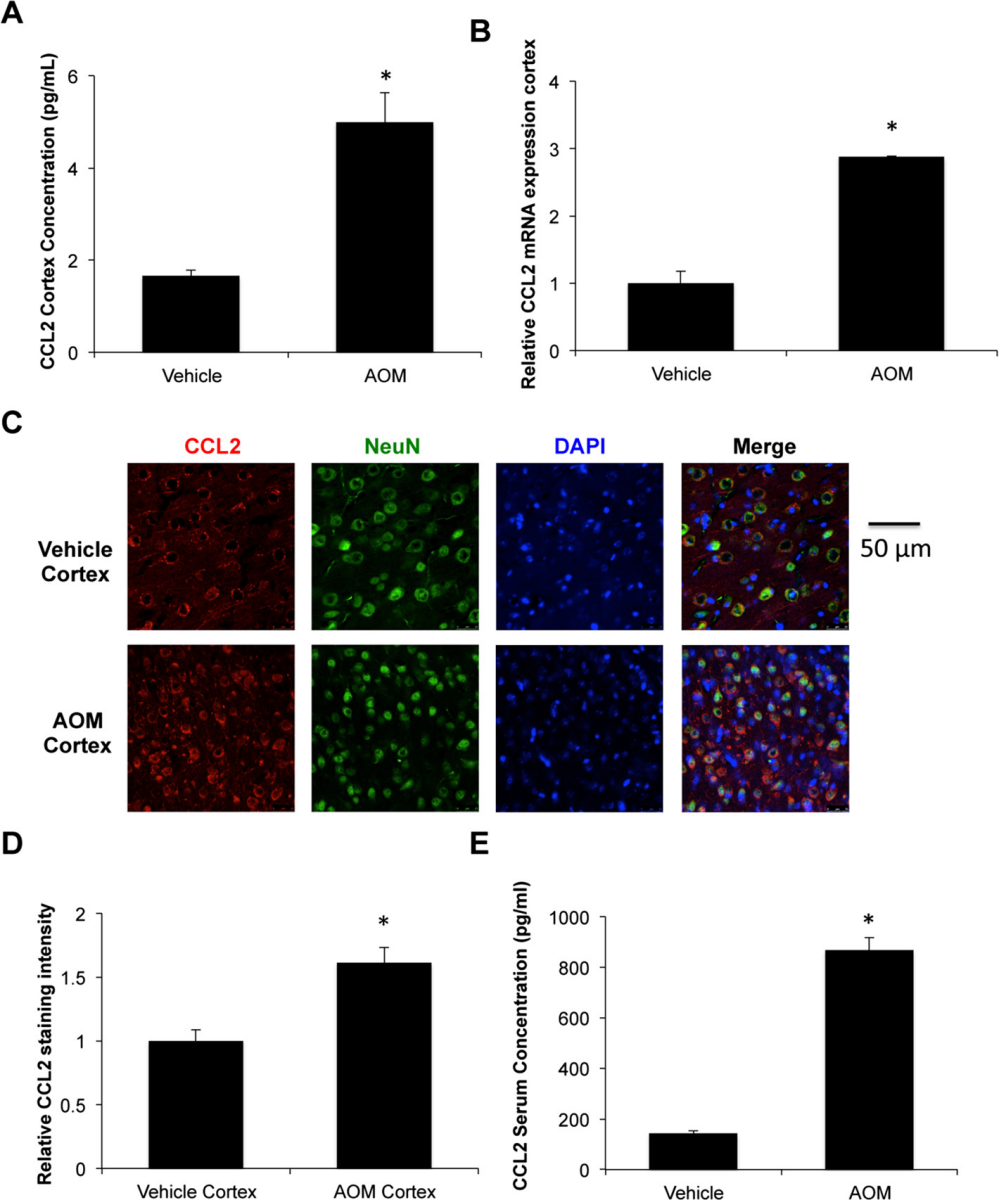
**CCL2 levels are elevated following AOM-induced liver failure. (A)** CCL2 concentrations in cortex lysates from vehicle and AOM-treated mice, n = 3. **(B)** CCL2 mRNA expression in the cortex of vehicle- and AOM-treated mice, n = 3. **(C)** Immunofluorescence for CCL2 (red) and NeuN (green) with DAPI (blue) used as a counterstain in vehicle- and AOM-treated mouse cortices. **(D)** Quantification of CCL2 immunofluorescence in vehicle- and AOM-treated mouse cortex, n = 3. **(E)** Serum levels of CCL2 in vehicle- and AOM-treated mice, n = 4. For ELISA, mRNA, and fluorescence quantification analyses * *P* <0.05 compared to vehicle-treated mice.

In order to determine if microglia activation was correlated with increased chemokine expression, neural CCL2 levels were measured. In the cortex, we observed a significant increase of CCL2 protein in AOM-treated mice (Figure 2A). In order to see if local increases in mRNA expression were driving these increases in protein, RT-PCR was performed. Expression of CCL2 mRNA was significantly upregulated in the cortex of AOM-treated mice compared to vehicle-treated mice (Figure 2B). CCL2 immunofluorescence was found to colocalize with the neuronal marker NeuN, indicating that CCL2 is expressed predominantly in neurons (Figure 2C). Quantification of this immunofluorescence demonstrated that AOM-treated mice had significantly increased CCL2 immunostaining in the cortex compared to vehicle-treated mice (Figure 2D). Outside of the brain, circulating levels of CCL2 were elevated in AOM-treated mice compared to vehicle-treated mice (Figure 2E).

### Inhibition of CCR2 or CCR4 is protective from AOM-induced neurological decline

**Figure 3:**
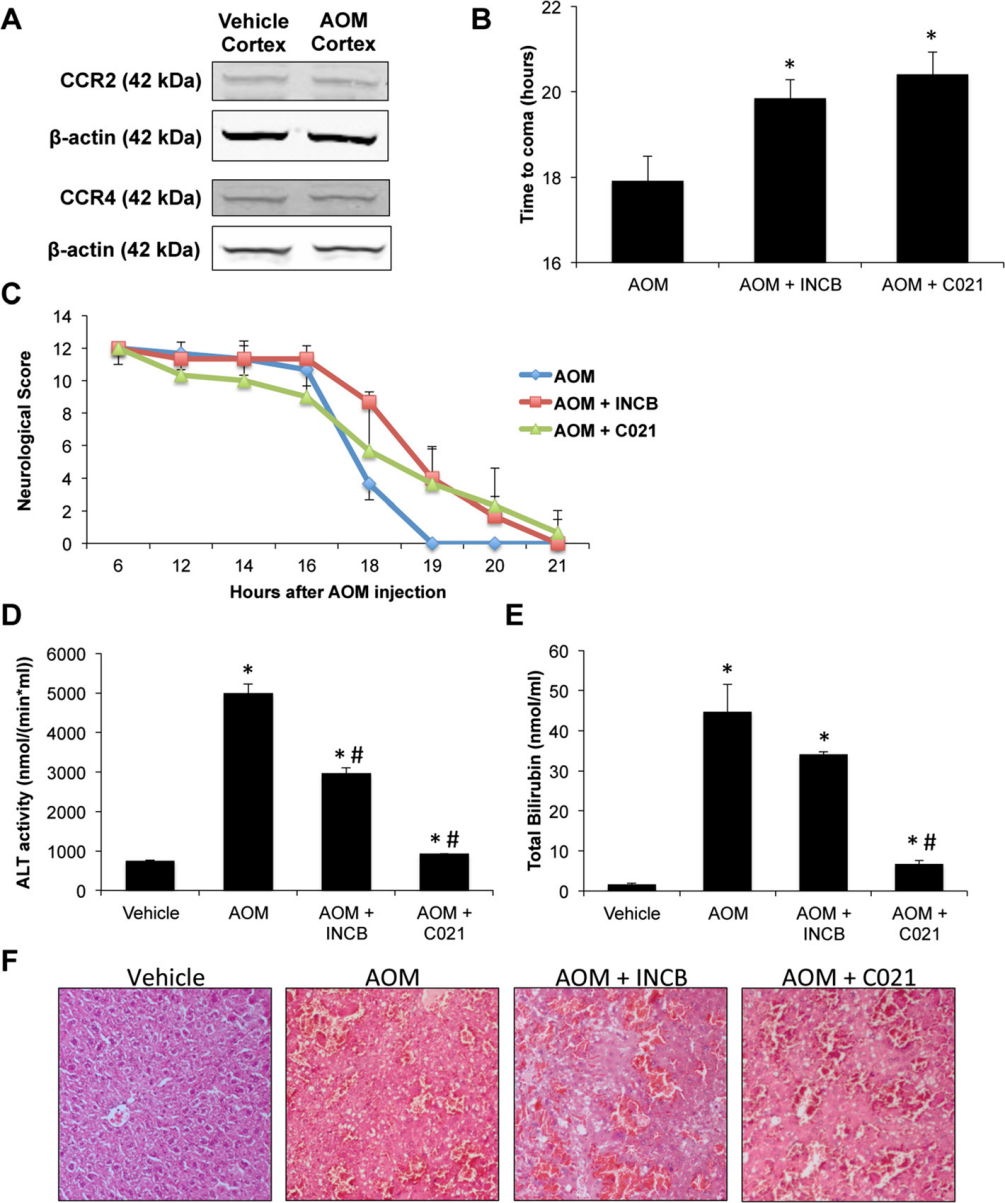
**CCR2 and CCR4 signaling play a role in the progression of hepatic encephalopathy. (A)** Representative immunoblots against CCR2 and CCR4 in vehicle- and AOM-treated cortex lysates. **(B)** Time to coma, in hours, of mice treated with AOM, AOM + INCB, or AOM + C021, n = 4. **(C)** Neurological score of mice treated with AOM, AOM + INCB, or AOM + C021, n = 4. Neurological score calculations are outlined in the methods section. **(D)** Serum ALT levels in mice treated with vehicle, AOM, AOM + INCB, or AOM + C021. **(E)** Bilirubin concentrations in serum for mice administered vehicle, AOM, AOM + INCB, or AOM + C021. **(F)** Hematoxylin and eosin stains in liver sections from mice treated with vehicle, AOM, AOM + INCB, or AOM + C021. For time to coma analyses, * *P* <0.05 compared to AOM-treated mice. For ALT and bilirubin assays, * *P* <0.05 compared to vehicle-treated mice, # *P* <0.05 compared to AOM-treated mice.

As CCL2 concentrations were systemically upregulated, the function of CCL2/CCR2 and CCL2/CCR4 receptor activity in this model was investigated. The protein expression of CCR2 and CCR4 was found to be unchanged in the cortex following AOM injection (Figure 3A). To assess the role of CCL2/CCR2 or CCL2/CCR4 signaling in the neurological complications of acute liver failure, antagonists against these receptors were injected prior to AOM administration. Pretreatment with either antagonist was found to significantly delay the neurological decline and increase the time taken to reach coma, indicating a neuroprotective effect of these treatments (Figure 3B,C). Analyses of liver enzymes after pretreatment with CCL2 receptor antagonists in AOM-treated mice demonstrated a significant reduction in the levels of ALT levels (Figure 3D) and bilirubin (Figure 3E) compared to AOM-treated mice alone, though levels were still.significantly elevated compared to controls indicating the presence of significant liver damage. This persistence of liver damage is evident in hematoxylin and eosin-stained liver sections, which indicate that significant necrosis and steatosis is still present in mice pretreated with either INCB or C021 (Figure 3F). Thus, it appears that even though significant liver damage was still present, pretreatment with INCB or C021 prior to AOM administration improved liver function.

### CCR2 and CCR4 inhibition reduces microglia activation in AOM-treated mice

**Figure 4:**
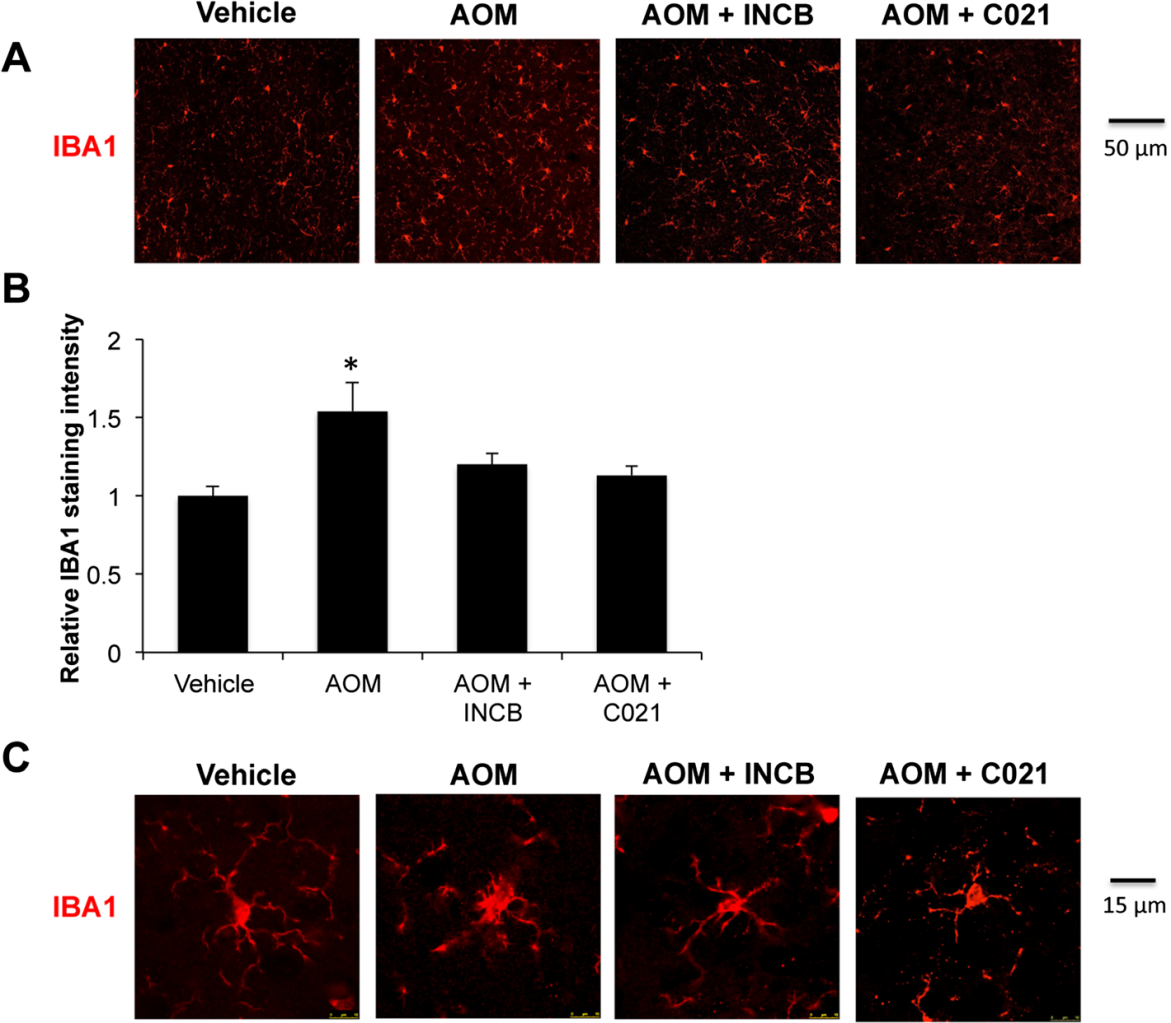
**CCR2 and CCR4 inhibition reduces microglia activation. (A)** Cortical IBA1 field fluorescence (red) in vehicle-, AOM-, AOM + INCB-, and AOM + C021-treated mice. **(B)** Quantification of IBA1 field fluorescence in vehicle-, AOM-, AOM + INCB-, and AOM + C021-treated mice, n = 3. **(C)** IBA1 immunofluorescence (red) in individual cells in the cortex of vehicle-, AOM-, AOM + INCB-, and AOM + C021-treated mice. For quantitative immunofluorescence analyses * *P* <0.05 compared to vehicle-treated mice.

Since CCR2 and CCR4 antagonism were found to reduce the neurological decline associated with AOM-induced liver damage, the effects of INCB and C021 on microglia proliferation and activation were assessed. Measurement of IBA1 field fluorescence identified that mice treated with INCB or C021 had significantly less microgliosis compared to mice treated with AOM alone (Figure 4A,B). Assessment of microglia morphology in mice treated with vehicle, AOM, AOM + INCB, and AOM + C021 identified that treatment of mice with the antagonists against CCR2 and CCR4 prevented the full activation of microglia (Figure 4C). Nevertheless, both partially activated and quiescent microglia were observed in mice treated with AOM after administration of either receptor antagonist.

**Figure 5:**
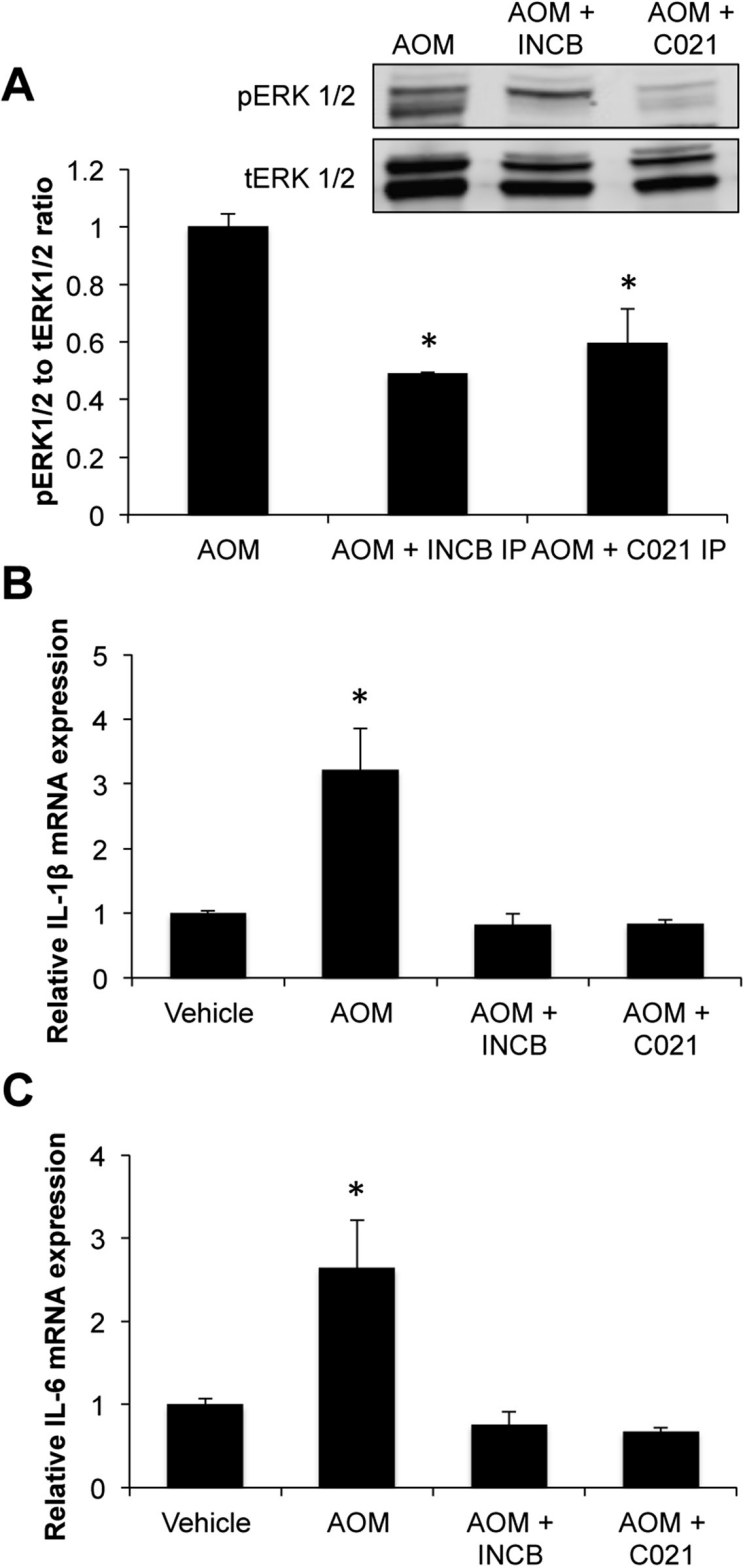
**INCB or C021 pretreatment reduces G-protein signaling pathway activity and proinflammatory cytokine production. (A)** Immunoblots and subsequent analyses for pERK1/2 and tERK1/2 in cortex lysates from mice administered AOM, AOM + INCB, and AOM + C021. **(B)** IL-1β mRNA expression in the cortex of vehicle-, AOM-, AOM + INCB-, and AOM + C021-treated mice, n = 3. **(C)** Cortical IL-6 mRNA expression in vehicle-, AOM-, AOM + INCB- and AOM + C021-treated mice, n = 3. For pERK1/2 immunoblotting analyses * *P* <0.05 compared to AOM-treated mice. For mRNA analyses * *P* <0.05 compared to vehicle-treated mice.

As treatment with both INCB and C021 was able to reduce microgliosis, validation that these treatments were reducing CCL2-dependent signaling was required. Other studies have shown that CCL2-mediated signaling leads to the phosphorylation of ERK1/2, which can be used as a measure of CCL2 downstream signaling [15]. Treatment with INCB or C021 was able to significantly reduce the pERK1/2 to tERK1/2 ratio (Figure 5A) compared to mice treated with AOM alone, indicating that these treatments are able to reduce downstream CCL2-mediated signaling. In order to see if this led to functional changes in neuroinflammation, RT-PCR was performed in the cortex of AOM-treated mice pretreated with C021 and INCB, which determined that IL-1β and IL-6 mRNA expression were elevated in AOM-treated mice, effects that were attenuated in mice pretreated with INCB or C021 (Figure 5B,C). Together, these data indicate that CCR2 and CCR4 signaling are required for microglia activation and subsequent production of the proinflammatory cytokines IL-1β and IL-6 following AOM-induced liver failure.

## Discussion

The major findings of this study relate to the role that CCL2 signaling plays in microglia activation and the subsequent proinflammatory response that occurs during hepatic encephalopathy. The findings from this study demonstrate that i) CCL2 activation occurs during AOM-induced neurological decline and mirrors microglia activation, and ii) inhibiting receptor activity of CCR2 or CCR4 reduces microglia activation, proinflammatory cytokine expression, and neurological decline in mice with hepatic encephalopathy. These findings suggest that increased CCL2/CCR2 and CCL2/CCR4 signaling, which is present during hepatic encephalopathy, leads to microglia activation and significantly contributes to the proinflammatory response observed. Thus, reducing CCL2 expression or inhibiting CCR2 or CCR4 activity may be potential treatment modalities for the management of hepatic encephalopathy.

Microglia activation has been demonstrated in various rodent models of hepatic encephalopathy, including hepatic devascularization [14], toxic liver injury [7], and biliary cirrhosis [16]. Furthermore, microglia activation has been observed in hepatic encephalopathy patients, including cirrhotic patients who died from hepatic coma [17], alcoholic patients that went on to develop hepatic encephalopathy [18], and in a viral hepatitis patient who developed hepatic encephalopathy [1]. Typically, microglia activation during hepatic encephalopathy is thought to be caused by elevations of cranial ammonia, lactate, glutamate, manganese, and neurosteroid concentrations [19]. Treatments that reduce microglia activation and are anti-inflammatory, such as the tumor necrosis factor inhibitor etanercept and therapeutic hypothermia, have been demonstrated to be protective against hepatic encephalopathy progression [20,21]. The results from the current study support the hypothesis that acute liver failure leads to microglia activation in the brain.

Previous research has demonstrated that CCL2 is expressed by a variety of cells in the CNS including neurons, microglia, endothelial cells, and astrocytes and can induce monocyte recruitment into the CNS [22-24]. Our studies identified that, during hepatic encephalopathy, CCL2 is elevated in the brain primarily in neurons. CCL2 elevation in the brain has been demonstrated in the bile duct ligation model of peripheral organ inflammation, where CCL2 or CCR2 knockout were able to reduce brain monocyte populations compared to wild-type, though upregulation of neural CCL2 occurred prior to infiltration of peripheral immune cells [16]. This study describes what occurs in the brain following chronic liver injury, but the specific effects of CCL2 in the brain following hepatic encephalopathy due to acute liver injury are not known. However, CCL2 has been implicated in other models of acute neuroinflammation. For instance, in a mouse model of controlled cortical impact, a transient upregulation of CCL2 occurred in the cortex at 24 h, returning to normal levels within 1 week [25]. Interestingly, in a model of intracranial hemorrhage, CCL2 knockout mice have reduced microglia activation only during the first 24 h, after which the levels are similar to wild-type mice [26]. Thus, it appears that CCL2 could be involved with acute microglia activation similar to that observed during AOM-induced hepatic encephalopathy, though chronic activation of microglia may be dependent upon other mechanisms.

In addition to increased brain CCL2 expression, our data indicate an elevation of CCL2 in the circulation during the neurological complications of acute liver failure. Elevated circulating levels of CCL2 have previously been shown in a number of other neuroinflammatory disorders such as ischemic stroke [27], Alzheimer’s disease [28], and traumatic brain injury [29]. The source of the CCL2 in the serum during hepatic encephalopathy and in other neuroinflammatory disorders is unknown. In the AOM model of hepatic encephalopathy it is conceivable that CCL2 may be peripherally or centrally derived. Indeed, elevation of hepatic CCL2 following liver damage has previously been demonstrated in alcoholic liver disease [30], non-alcoholic steatohepatitis [31], and in acute liver failure [32]. Furthermore, CCL2 contributes to liver damage and strategies to inhibit CCL2 expression or function appear to be protective in a number of models of liver injury [33,34].

Here, we demonstrate that inhibition of CCL2 signaling in the liver using CCR2 and CCR4 antagonists is somewhat hepatoprotective. The data supports that the significant parenchymal damage that occurs in AOM mice still persists following INCB or C021 treatment, though liver function is improved as measured by both serum ALT and bilirubin levels. In support of our findings, CCR2 knockout mice are resistant to hepatitis and have reduced infiltration of inflammatory monocytes [35]. Conversely, studies using an acetaminophen model of liver injury identified that CCR2 knockout exacerbated liver injury [36]. Thus, it appears that CCR2-dependent signaling in the liver is context dependent. Little information exists regarding the specific role for CCR4 in models of liver damage. Interestingly, macrophages and other immune cells are regulated by both CCR2 and CCR4 and thus it is possible that reducing their activity may prevent immune cell recruitment to the liver and subsequent acute injury.

It is conceivable that the source of CCL2 that contributes to the neurological decline in AOM-treated mice may be peripherally-derived, but for this to be reasonable, CCL2 must be able to cross the blood-brain barrier (BBB). Interestingly, the BBB is disrupted in AOM-treated mice, which leads to vasogenic edema [37]; however, the mechanism by which the BBB is permeabilized is not clearly defined. CCL2 has been previously shown to exert an effect on BBB permeability in a number of models of neuroinflammation. For instance, in a model of stroke CCL2 knockout mice have decreased BBB permeability compared to wild-type mice [38]. Also, *in vitro* models of the BBB have demonstrated that CCL2 helps promote permeability of brain endothelial cell and astrocyte bilayers [39]. Other studies have found that CCL2 can undergo transcellular transport across intact endothelial cell monolayers, giving support that systemic CCL2 may have the capability to generate neuroimmunological effects in the absence of overt changes in BBB permeability [40]. The direct contribution of CCL2 on BBB permeability and cerebral edema following acute liver failure is unknown; however, other studies have shown that microglia are able to recruit circulating monocytes into the brain following bile duct resection, a model that can be used to generate hepatic encephalopathy due to chronic liver injury, and that this recruitment was dependent on both CCL2 and CCR2 [16]. In the current study, systemic treatment with INCB and C021 generated effects on both the liver and brain and this could be due to the improvement of liver function and a subsequent decrease in toxic metabolites that enter the brain, a reduction of circulating immune cell recruitment to the brain, or reduction of cerebral edema. Ongoing studies are being performed to address the specific mechanisms that INCB and C021 generate their neuroprotective effects during hepatic encephalopathy.

In humans, microglia have been demonstrated to express a variety of chemokine receptors, including CCR1, CCR2, CCR4, CCR5, CXCR1, CXCR3, and CX3CR1, indicating that their function can be regulated by a variety of chemokines [41]. Even though CCR2 and CCR4 are the primary receptors for CCL2, it is possible that INCB and C021 could inhibit the activity of other chemokines. There are recent reports of CCL2 binding at chemokine receptor-independent sites, such as the D6 decoy chemokine receptor, which leads to sequestration of this chemokine [42]. This has been demonstrated in primary adult human astrocytes where CCL2 is able to bind astrocytes that do not express CCR2 but do express the D6 decoy chemokine receptor [43]. Thus, it may be possible that shifts in CCL2/CCR2/CCR4 activity, along with activities of other chemokines and receptors, play important roles in the neurological decline associated with hepatic encephalopathy, though further studies in this area are still warranted.

## Conclusions

The results presented here suggest that, following acute liver failure, microglia activation via CCL2-induced signaling contributes to the neurological decline associated with hepatic encephalopathy. Our results suggest that modulating systemic CCL2 signaling via the use of INCB or C021 to inhibit CCR2 and CCR4 helps improve liver function, leads to reduced microglia activation, and reduces neurological decline compared to mice treated with AOM alone. Therefore, treatments aimed at reducing circulating levels of CCL2, or reducing the activity of CCR2 or CCR4, may be a potential treatment paradigm for patients with hepatic encephalopathy.

## Abbreviations

ALT: Alanine aminotransferase; AOM: Azoxymethane; BBB: Blood-brain barrier; CCL2: Chemokine ligand 2; CCR2: Chemokine receptor 2; CCR4: Chemokine receptor 4, C021, C 021 dihydrochloride; IL: Interleukin; INCB: INCB 3284 dimesylate; ip: intraperitoneal; pERK1/2: Phosphorylated extracellular signal-regulated kinase 1/2; RT-PCR: Real-time PCR; tERK1/2: Total extracellular signal-regulated kinase 1/2.

## Competing interests

The authors have no competing interests to declare.

## Authors’ contributions

MM, GF, MB, CG, HS, and EW performed technical work and data analysis. MM, GF, and SD performed statistical analyses. MM, GA, and SD conceived of the study, designed and coordinated experiments, and drafted the manuscript. All authors have read and approved the final manuscript.
